# Cost-effectiveness analysis of revisional Roux-en-Y gastric bypass: laparoscopic vs. robot assisted

**DOI:** 10.1007/s13304-022-01425-z

**Published:** 2022-11-23

**Authors:** Elettra Ugliono, Fabrizio Rebecchi, Costanza Vicentini, Antonio Salzano, Mario Morino

**Affiliations:** 1 Department of Surgical Sciences, University of Turin, Corso A.M. Dogliotti 14, 10126 Turin, Italy; 2 Department of Mechanical and Aerospacial Engineering, Politecnico of Turin, Corso Duca Degli Abruzzi 24, 10129 Turin, Italy; 3 Department of Public Health and Pediatrics, University of Turin, Via Santena 5 Bis, 10126 Turin, Italy

**Keywords:** Bariatric surgery, Roux-en-Y gastric bypass, Revisional surgery, Cost-effectiveness, Cost-utility, Cost analysis

## Abstract

There is controversy over the possible advantages of the robotic technology in revisional bariatric surgery. The aim of this study is to report the experience of a high-volume bariatric center on revisional Roux-en-Y gastric bypass with robot-assisted (R-rRYGB) and laparoscopic (L-rRYGB) approaches, with regards to operative outcomes and costs. Patients who underwent R-rRYGB and L-rRYGB between 2008 and 2021 were included. Patients’ baseline characteristics and perioperative data were recorded. The primary endpoint was the overall postoperative morbidity. A full economic evaluation was performed. One-way and two-way sensitivity analyses were performed on laparoscopic anastomotic leak and reoperation rates. A total of 194 patients were included: 44 (22.7%) L-rRYGB and 150 (77.3%) R-rRYGB. The robotic approach was associated with lower overall complication rate (10% vs. 22.7%, *p* = 0.038), longer operative time, and a reduced length of stay compared to L-rRYGB. R-rRYGB was more expensive than L-rRYGB (mean difference 2401.1€, *p* < 0.001). The incremental cost-effective ratio (ICER) was 18,906.3€/complication and the incremental cost-utility ratio was 48,022.0€/QALY (quality-adjusted life years), that is below the willingness-to-pay threshold. Decision tree analysis showed that L-rRYGB was the most cost-effective strategy in the base-case scenario; a probability of leak ≥ 13%, or a probability of reoperation ≥ 14% following L-rRYGB, or a 12.7% reduction in robotic costs would be required for R-rRYGB to become the most cost-effective strategy. R-rRYGB was associated with higher costs than L-rRYGB in our base-case scenario. However, it is an acceptable alternative from a cost-effectiveness perspective.

## Introduction

The need for revisional bariatric surgery (RBS) following primary bariatric surgery has risen rapidly during the last decades and is expected to grow further. RBS procedures are technically complex and associated with higher morbidity and mortality rates than primary operations [1, 2].

The use of the DaVinci robotic platform (Intuitive Surgical Inc., Sunnyvale, CA, USA) has been advocated for advanced surgical procedures in light of the advantages offered by this technology, such as the three-dimensional stereoscopic imaging and the enhanced operative dexterity with seven degrees of freedom of the EndoWrist robotic instruments.

There are limited published data concerning robotic RBS, consisting mainly of small single-center series, with a wide heterogeneity of indications and types of surgical procedures performed [3, 5]. Nevertheless, the clinical outcomes of robot-assisted RBS compare favorably with the laparoscopic series presented in the literature, suggesting a promising role of DaVinci in this field [6–8].

The main criticism over robotic surgery is related to its costs; it has been suggested that the high expenditures of the robotic platform could be counterbalanced, to some extent, by the reduction in costly complication rates [9]. However, no previous economic evaluation assessing the cost-effectiveness of the robotic approach in RBS has been published.

This study aims to report the experience of a high-volume tertiary academic center on the utilization of the robotic platform in performing revisional Roux-en-Y gastric bypass (RYGB), with respect to clinical and economic outcomes.

## Methods

We performed a full economic evaluation to compare revisional RYGB performed with laparoscopic (L-rRYGB) and robot-assisted (R-rRYGB) approaches. The input parameters integrated data from our institution with data from the published literature.

### Input data

#### Data on clinical effectiveness and complications

Patients who underwent R-rRYGB and L-rRYGB between 2008 and 2021 were included.

Inclusion criteria were conversions from adjustable gastric banding, vertical banded gastroplasty, and sleeve gastrectomy for insufficient weight loss, weight regain, or complications of the index procedure (dysphagia, gastroesophageal reflux disease). Exclusion criteria were acute complications requiring emergency surgery and revisional procedures performed with the open approach.

All the procedures were performed by the same two proficient surgeons with more than 20-year experience both in laparoscopic and in robot-assisted surgery. The detailed descriptions of the surgical techniques were previously published [10, 11]. Patients underwent L-rRYGB or R-rRYGB depending on the economical availability of the robotic system, and not to clinical characteristics of the patients. Our institution progressively increased the financial resources allocated to robot-assisted surgery, leading us to shift towards the exclusive use of the robotic platform in complex surgeries such as RBS, that has become the conventional approach since 2010. We collected data regarding preoperative patients’ characteristics, type of primary procedure, indications for reoperation, perioperative data, and postoperative complications. Major complications were defined as a value ≥ 3 according to the Clavien–Dindo classification [12].

#### Data on resource utilization and cost

The economic assessment included costs related to operative room time, length of stay, surgical tools, and robotic system maintenance, that were provided by the Institution’s financial department. Table 1 summarizes the costs of major resources analyzed.

**Table 1:** Unit costs of major resources analyzed

Parameter	Unit	Unit cost indexed for 2021	Source
Operating room	Hour		IFD
Laparoscopic		320 €	
Robotic		610 €	
Hospital stay	Day		IFD
Surgical ward		560 €	
Intensive care unit		2000 €	
Laparoscopic tools	Procedure		IFD
Circular stapler		153.7 €	
Linear stapler		384.3€	
Cartridges		164.7€	
Robotic tools	Procedure	2413.0 €	IFD
Robotic system maintenance	Month	18,807.5 €	IFD
QALYs			
Initial procedure-related decrement	QALYs	− 0.22	Campbell et al. [13]
Utility decrement minor complication		− 0.11	
Utility decrement major complication		− 0.22	
Utility decrement reoperation		− 0.36	

*IFD* institution financial department, *QALYs* quality-adjusted life years

We did not include the expenditures related to health-care personnel, drugs, and diagnostics (laboratory tests and radiological examinations) that we estimated to be comparable between the two approaches. All the costs were real values, with 22% VAT included when applicable. Purchase of the robotic platform was not considered. All costs in this analysis were evaluated in Euros (€).

#### Health-related quality of life

Health-related quality of life data were calculated using utility values published in the literature [13]. Utility decrements, estimated with the Euro-Qol-5D, were dependent on the initial RBS and on the occurrence of postoperative complications (Table 1).

#### Statistical analysis and economic evaluation

Quantitative data are given as mean and standard deviation, categorical data as percentages, and were compared using the *t* test and *c*^2^ test, respectively. A 2-sided *p* value < 0.05 was considered statistically significant. The statistical analyses were performed using SPSS software.

The economic evaluation was performed from a hospital health-care perspective with a time horizon of 1 year. The cost-effectiveness endpoint was the cost associated with a unitary reduction in overall postoperative complications and the incremental cost-effectiveness ratio (ICER) was calculated.

The cost-utility endpoint was the cost per quality-adjusted life years (QALYs); R-rRYGB was considered cost-effective if the incremental cost-utility ratio (ICUR) was below the willingness-to-pay threshold of € 50,000/QALY [14].

A decision tree model was developed to compare the two strategies to evaluate the impact of anastomotic leaks and reoperations on economic outcomes. Patients undergo either R-rRYGB or L-rRYGB, and transition along the arms of the decision tree was according to the probability of three outcomes: uncomplicated procedure, anastomotic leak or reintervention. A cost value was assigned to every node in the sequence of events leading to an outcome. We calculated the mean cost of uncomplicated R-rRYGB and L-rRYGB, and we estimated the cost of an anastomotic leak and of a reoperation by subtracting the cost of uncomplicated cases from the total costs of complicated cases. Based on the likelihood of the sequence of events in each arm, a total cost per strategy was obtained. A strategy was considered cost-effective if associated with the lowest cost. A threshold value beyond which a strategy would be considered cost-effective compared to the alternative strategy was determined. The decision tree model was created using SilverDecisions [15].

One-way and two-way sensitivity analyses were performed on (1) the probability of anastomotic leak, (2) the probability of reintervention in the laparoscopic surgery arm and (3) the reduction in costs for uncomplicated robotic cases. The results of the study are reported according to the CHEERS guidelines [16].

## Results

### Clinical results

From 2008 to 2021, 194 patients were included: 44 (22.7%) L-rRYGB and 150 (77.3%) R-rRYGB. Patients’ baseline characteristics are presented in Table 2.

**Table 2:** Demographic and baseline clinical characteristics of patients

	R-rRYGB *N* = 150	L-rRYGB *N* = 44	*P*
Mean age (years)	49.0 ± 10.1	48. 0 ± 10.5	1.0
Gender (*n*, %)			
Male	12 (8.0%)	11 (25.0%)	0.006
Female	138 (92.0%)	33 (75.0%)	
Mean weight (kg)	94.2 ± 23.1	105.2 ± 25.4	0.007
Mean BMI (kg/m^2^)	35.6 ± 7.2	38.0 ± 8.5	0.06
Comorbidities			
Hypertension	51 (34.0%)	18 (40.9%)	0.47
Diabetes	6 (4.0%)	6 (13.6%)	0.03
OSAS	13 (8.7%)	4 (9.1%)	1.0
Dyslipidemia	6 (4.0%)	2 (4.5%)	1.0
Esophagogastroduodenoscopy			
Esophagitis	46 (30.7%)	8 (18.2%)	0.12
HP infection	8 (5.3%)	0 (0%)	0.20
Hiatal hernia	37 (24.7%)	11 (25.0%)	1.0
Barrett’s esophagus	1 (0.7%)	0 (0%)	1.0
Index bariatric procedure			
Adjustable gastric banding	27 (18%)	8 (18.2%)	1.0
Vertical banded gastroplasty	74 (49.3%)	31 (70.4%)	0.016
Sleeve gastrectomy	49 (32.7%)	5 (11.4%)	0.007
Indication for revisional surgery			
Insufficient weight loss	16 (10.7%)	7 (15.9%)	0.42
Weight regain	47 (31.3%)	18 (40.9%)	0.27
Gastroesophageal reflux	38 (25.3%)	5 (11.4%)	0.06
Dysphagia	49 (32.6%)	14 (31.8%)	1.0

*R-rRYGB* robotic revisional Roux-en-Y gastric bypass, *L-r-RYGB* laparoscopic revisional Roux-en-Y-gastric bypass, *BMI* body mass index, *OSAS* obstructive sleep apnea syndrome, *HP* helicobacter pylori

R-rRYGB was associated with longer operative time (253.1 ± 50.4 vs. 207.8 ± 66.2 min, *p* = 0.0001) and lower overall postoperative complications (10% vs. 22.7%, *p* = 0.038) than L-rRYGB.

In R-rRYGB, there were 9 (6.0%) minor and 6 (4.0%) major complications: 3 pulmonary complications requiring ICU stay, 1 bleeding requiring endoscopic hemostasis, 1 (0.6%) anastomotic leak treated conservatively, and 1 reintervention (0.6%) for small bowel perforation. In L-rRYGB, there were 4 (9.1%) minor and 6 (13.6%) major complications: 3 (6.8%) anastomotic leaks (1 requiring reintervention), 2 intestinal obstructions submitted to surgical revision, and 1 anastomotic bleeding requiring endoscopic hemostasis.

The mean length of stay was significantly shorter for the robotic approach (5.2 ± 4.7 vs. 7.2 ± 6.3 days, *p* = 0.018). Mortality was 0% for R-RYGB and 2.3% for L-rRYGB (*p* = 0.22).

### Overall costs

**Fig. 1:**
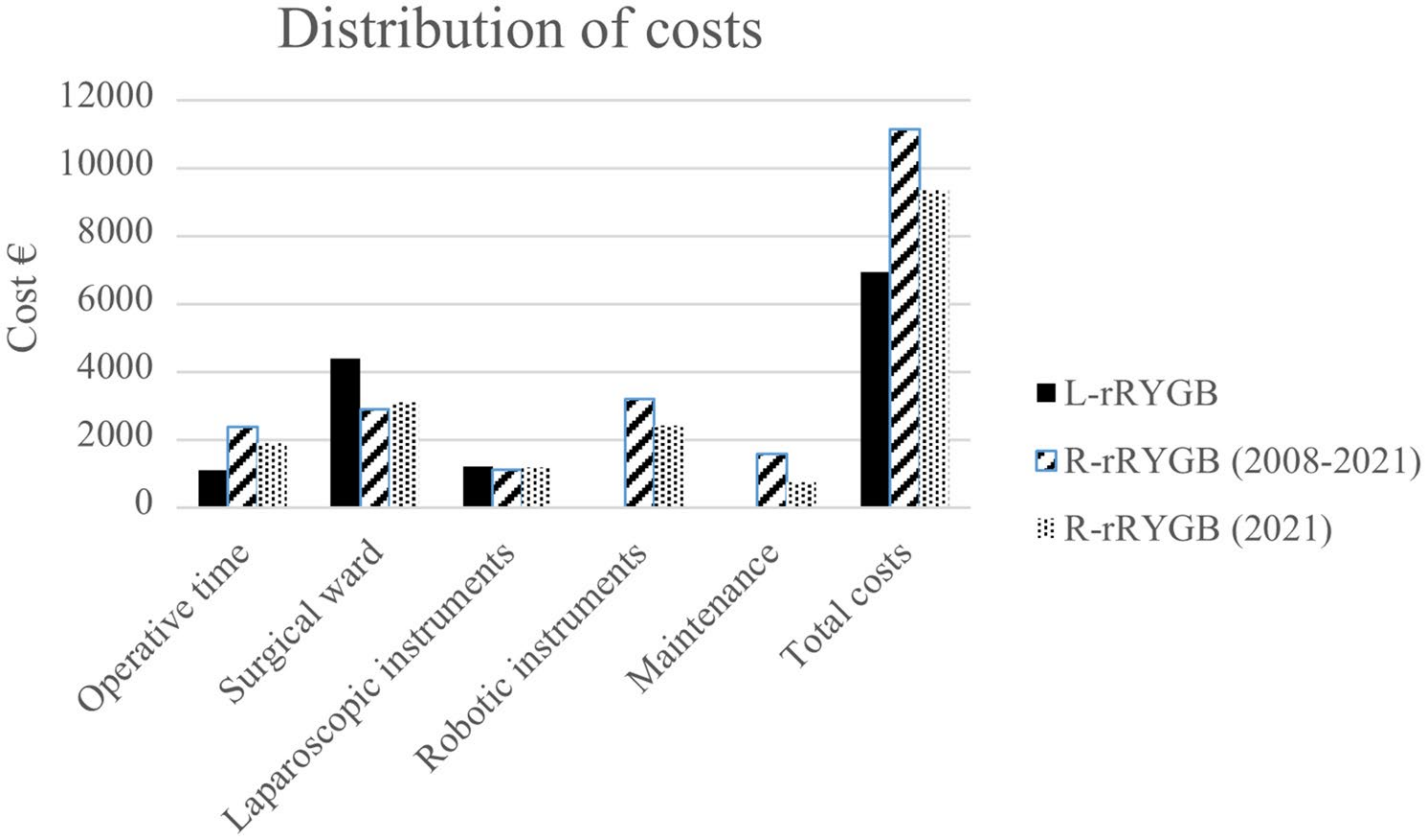
Distribution of costs of laparoscopic and robot-assisted revisional Roux-en-Y gastric bypass

**Fig. 2:**
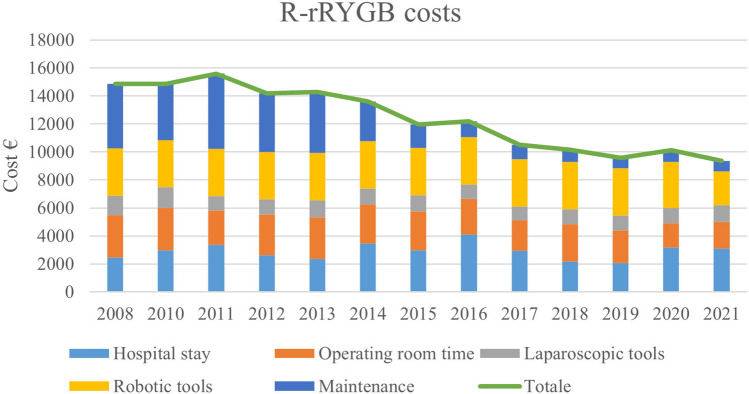
Yearly mean overall costs per R-rRYGB procedure and proportion of costs of single components. *R-rRYGB* Robotic revisional Roux-en-Y gastric bypass

The mean overall costs were 6956.6 ± 4547.8 € for L-rRYGB and 11,151.2 ± 3225.8 € for the entire robotic series (mean difference 4194.6€, *p* < 0.001). Considering only R-rRYGB procedures performed in 2021, the mean overall costs was 9357.8 ± 4339.2 € (mean difference 2401.2€, *p* < 0.001). Figure 1 shows the differences in hospital health-care costs. In fact, there was a progressive reduction of R-rRYGB costs, from 14,851.0 ± 324.7 € in 2008 to 9357.8 ± 4369.2 € in 2021 (*p* = 0.009). This reduction was mainly driven by a reduction in the expenditures related to robotic system maintenance, robotic semi-disposable tools and operative room time. Figure 2 shows overall costs per surgical procedure and the proportion of the single components.

### Cost-effectiveness and cost-utility analyses

R-rRYGB was associated with increased costs compared to L-rRYGB by 2401.1€, and the ICER was 18,906.3 €/complication. In the base-case analysis, R-rRYGB generated an additional 0.05 QALYs, resulting in an ICUR of 48,022.0€/QALY, that is below the willingness-to-pay threshold (Table 3).

**Table 3:** Base-case results of cost-effectiveness and cost-utility analyses

	Costs	Δ costs	Effectiveness	Δ Effectiveness	ICER	QALYs	Δ QALYs	ICUR
R-rRYGB	9357.8 €	2401.1 €	90%	12.7%	18,906.3 €/complication	0.76	0.05	48,022.0€/QALY
L-rRYGB	6956.7 €		77.3%			0.71		

*Δ* difference, *ICER* incremental cost-effective ratio, *QALYs* quality-adjusted life years, *ICUR* incremental cost-utility ratio

### Decision tree analysis and sensitivity analysis

**Fig. 3:**
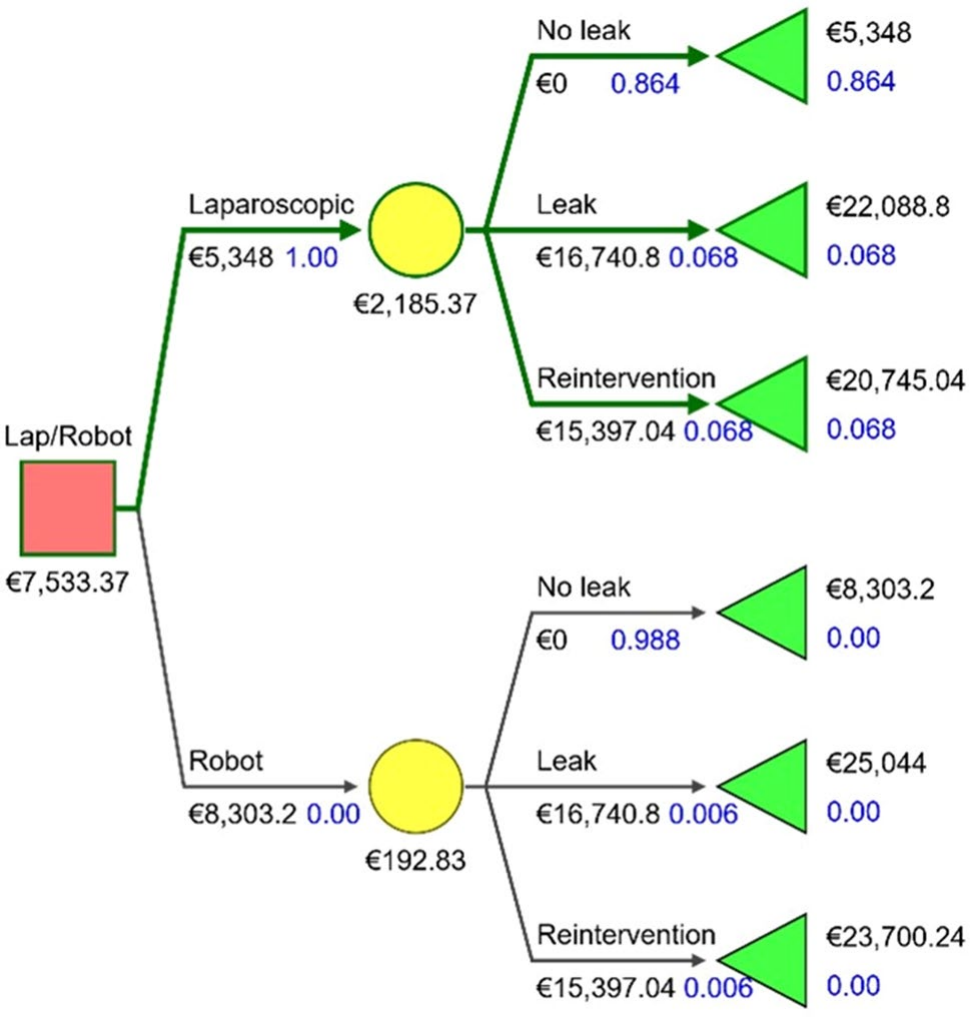
Decision tree model for base-case scenario

Input parameters for decision tree analysis are summarized in Table 4. L-rRYGB was the most cost-effective strategy in the base-case scenario (7533.4€ vs. 8496.0€), and the mean difference was 962.6 € (Fig. 3).

**Table 4:** Decision tree input parameters

	R-rRYGB	L-rRYGB
Cost of uncomplicated procedure	8303.2 ± 800.4 €	5348.0 ± 1339.6
Cost of leak	16,740.8 €	16,740.8 €
Leak rate (%)	0.60%	6.80%
Cost of reoperation	15,397.0 €	15,397.0 €
Reoperation rate (%)	0.60%	6.80%

*R-rRYGB* Robot-assisted revisional Roux-en-Y gastric bypass, *L-rRYGB* laparoscopic revisional Roux-en-Y gastric bypass

**Fig. 4:**
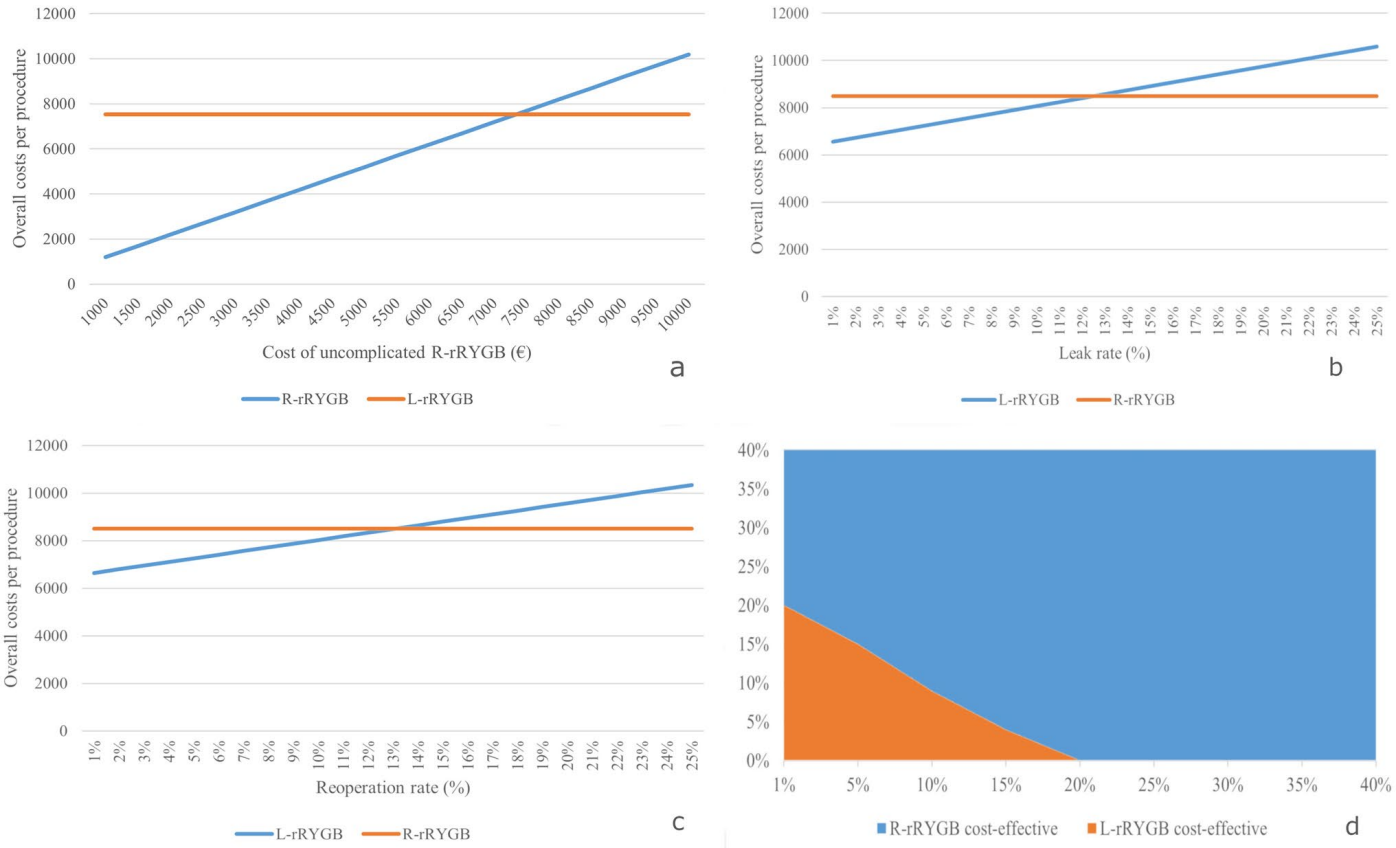
Results of one-way and two-way sensitivity analyses. **a**–**d** The results of one-way and two-way sensitivity analyses. **a** One-way sensitivity for uncomplicated robot-assisted revisional Roux-en-Y gastric bypass (R-rRYGB) costs; **b** one-way sensitivity for laparoscopic revisional Roux-en-Y gastric bypass (L-rRYGB) anastomotic leak rate; **c** one-way sensitivity for L-rRYGB reoperation rate; **d** two-way sensitivity analysis for L-rRYGB anastomotic leak and reoperation rates

R-rRYGB was more cost-effective than L-rRYGB when the cost of uncomplicated robotic cases dropped under 7250.0 € (− 1053.2€ compared to base-case input value) while maintaining fixed leak and reoperation rates. A probability of leak following L-rRYGB of over 13% or a probability of reoperation following L-rRYGB of over 14% would be required for R-rRYGB to become the most cost-effective strategy. According to the results of two-way sensitivity analysis, when the probabilities of both complications were low, L-rRYGB was preferred; as the probabilities increased, R-rRYGB was preferred. Figure 4 shows the results sensitivity analyses.

## Discussion

RBS are technically complex procedures, associated with higher risks of postoperative complications, reoperations, and mortality than primary procedures [1, 2]. The robotic technology, overcoming some of the limitations of laparoscopy, such as the possibility to easily perform hand-sewn intracorporeal anastomosis and multiple quadrant access, could offer advantages, especially in complex surgical procedures.

There is controversy concerning the role of the robotic platform in bariatric surgery. Some systematic reviews and meta-analyses have shown a lower incidence of anastomotic complications (leak and strictures) associated with the use of robotic technology than the laparoscopic approach when performing primary RYGB [17–19]. However, others did not confirm these results [20, 21]. Concerning the application of the robotic platform in RBS, the evidence in the literature is more limited. Several authors reported their experience on R-rRYGB with promising results, demonstrating the safety and feasibility of the procedure [3–5]. However, studies directly comparing R-rRYGB and L-rRYGB are scarce [6–8]. In fact, RBS are challenging procedures that should be performed only in selected highly experienced bariatric centers, where the robotic platform might not be available [22].

Recently, Bertoni et al. published a systematic review and meta-analysis including six non-randomized comparative studies including 2459 robotic and 27,431 laparoscopic RBS patients [23]. In a subgroup analysis of revisional RYGB, there were no significant differences in early postoperative complications (9.2% vs. 11.6% *p* = 0.12), reoperations, and length of stay. However, the study by Nasser et al., which accounted for 93% of the patients included in the metanalysis, did not directly compare L-rRYGB and R-rRYGB since data were obtained from a national clinical registry, and this database did not directly collect information regarding anastomotic leaks, which could be underestimated [24]. Furthermore, this metanalysis had strict inclusion criteria, as it included only comparative studies, and the results differ significantly from those of other systematic reviews and meta-analyses comparing primary and revisional RYGB, which have reported an overall morbidity ranging from 18.6 to 29.5%, a reoperation rate of 8.4% and an anastomotic leak rate of 4.3–5.8% after revisional RYGB, which are in line with our results for the laparoscopic series [1, 2].

In the present study, there was only one anastomotic leak in R-rRYGB group (0.6% vs. 6.8% of L-rRYGB, *p* = 0.037), which is the most fearsome complication and contributes significantly to morbidity and mortality after revisional RYGB. These favorable results could be explained by the better visualization and surgical dexterity offered by the robotic platform, that could lead to a more precise adhesion and tissue dissections and the possibility to perform hand-sewn gastro-jejunal anastomoses when limited gastric remnant is available, as in the context of previous bariatric procedures.

The main criticism addressed to robotic surgery is related to its high costs, due to the purchase and the maintenance of the robotic system, and expensive semi-disposable robotic instruments. In a previous study, we demonstrated a significant reduction in intraoperative costs per surgical procedure with the reduction in operative room time, the decreased use of laparoscopic staplers performing hand-sewn anastomoses, and the multidisciplinary use of the robotic system.

In the current study, we report a progressive decrease in overall costs per procedure. This reduction was mainly driven by the amortization of the maintenance costs, which accounted for more than 30% in 2008 and fell to less than 10% with the implementation of the multidisciplinary use of the robotic platform. The impact of robotic instruments remained stable over time thanks to a renegotiation of supplies in 2020 and with the launch of the Extended Use Program in 2021, ensuring an increased number of lives per semi-disposable robotic instrument.

We showed that R-rRYGB was associated with higher costs than L-rRYGB, with an ICER of 18,906.3 €/complication and an ICUR of 48,022.0€/QALY. Although an official threshold for the ICER does not exist in Italy, the ICUR values obtained were below the commonly accepted willingness-to-pay threshold of 50,000€/QALY [14].

We built a decision tree model to investigate whether the savings associated with the avoidance of complications of R-rRYGB would be enough to compensate for the reduced costs of L-rRYGB. We demonstrated that L-rRYGB was the most cost-effective strategy, and extremely high rates of anastomotic leaks or reinterventions would be necessary for R-rRYGB to become the preferred strategy. The base-case analysis showed that the expected average cost was 7533.4€ for L-rRYGB and 8496.0€ for R-rRYGB. Therefore, the overall difference between the two procedures was 962.6 €. According to the results of sensitivity analyses, a further reduction of 12.7% in robotic costs for uncomplicated cases would be required for R-rRYGB to become cost-effective over L-rRYGB.

To date, Intuitive Surgical (Sunnyvale, CA, USA) is the sole provider of robotic surgery. However, in the future, the entry of new competitors in the market will translate into lower pricing of the robotic equipment, which could change the perspectives on robotic-assisted procedures.

This study has limitations that should be considered. Data concerning R-rRYGB come from a high-volume specialized institution with extensive experience both with laparoscopic and robotic RBS, and the time period considered for the two approaches was different due to the progressive implementation of the robotic technology in our institution. We did not consider costs associated with the purchase of the robotic system since this value would differ significantly in different health-care services and thus reduce the generalizability of our results. The precision of our estimates of anastomotic leaks and reoperations costs is limited by the paucity of data, given the low frequency of such complications, and the heterogeneity of the clinical course associated with these conditions. Finally, data were collected retrospectively. However, we believe that the retrospective nature of the study has a limited impact on the results, since the objective was not to demonstrate the superiority of R-rRYGB over L-rRYGB, but to show that despite the higher costs, the robotic platform could be considered an acceptable alternative to laparoscopy for complex surgeries such as RBS.

## Conclusions

R-rRYGB is a safe and feasible procedure with a favorable safety profile compared to L-rRYGB. The costs associated with R-rRYGB are superior to L-rRYGB and remain such even considering a potential higher rate of anastomotic leaks and complications of the laparoscopic approach. However, R-rRYGB is an acceptable alternative from a cost-effectiveness perspective.

## Data Availability

Due to privacy and ethical concerns, supporting data are not available publicly.

## References

[1] Pędziwiatr M, Małczak P, Wierdak M, Rubinkiewicz M, Pisarska M, Major P, Wysocki M, Karcz WK, Budzyński A (2018). Revisional gastric bypass is inferior to primary gastric bypass in terms of short- and long-term outcomes-systematic review and meta-analysis. Obes Surg.

[2] Mahawar KK, Graham Y, Carr WR, Jennings N, Schroeder N, Balupuri S, Small PK (2015). Revisional Roux-en-Y gastric bypass and sleeve gastrectomy: a systematic review of comparative outcomes with respective primary procedures. Obes Surg.

[3] Rebecchi F, Ugliono E, Allaix ME, Toppino M, Borello A, Morino M (2020). Robotic Roux-en-Y gastric bypass as a revisional bariatric procedure: a single-center prospective cohort study. Obes Surg.

[4] Dreifuss NH, Mangano A, Hassan C, Masrur MA (2021). Robotic revisional bariatric surgery: a high-volume center experience. Obes Surg.

[5] Vilallonga R, Cirera de Tudela A, Möller EG, Piñeiro LV, Segura MB, Ferreruela MP, Mata RM, Caubet E, Gonzalez O, de Gordejuela AGR, Ciudin A, Fort JM, Carrasco MA (2021). Robotic revisional experience. Single centre prospective cohort study and review of the literature. Chirurgia (Bucur).

[6] Gray KD, Moore MD, Elmously A, Bellorin O, Zarnegar R, Dakin G, Pomp A, Afaneh C (2018). Perioperative outcomes of laparoscopic and robotic revisional bariatric surgery in a complex patient population. Obes Surg.

[7] Buchs NC, Pugin F, Azagury DE, Huber O, Chassot G, Morel P (2014). Robotic revisional bariatric surgery: a comparative study with laparoscopic and open surgery. Int J Med Robot.

[8] Beckmann JH, Mehdorn AS, Kersebaum JN, von Schönfels W, Taivankhuu T, Laudes M, Egberts JH, Becker T (2020). Pros and cons of robotic revisional bariatric surgery. Visc Med.

[9] Hagen ME, Pugin F, Chassot G, Huber O, Buchs N, Iranmanesh P, Morel P (2012). Reducing cost of surgery by avoiding complications: the model of robotic Roux-en-Y gastric bypass. Obes Surg.

[10] Rebecchi F, Ugliono E, Palagi S, Genzone A, Toppino M, Morino M (2021). Robotic "Double Loop" Roux-en-Y gastric bypass reduces the risk of postoperative internal hernias: a prospective observational study. Surg Endosc.

[11] Rebecchi F, Allaix ME, Ugliono E, Giaccone C, Toppino M, Morino M (2016). Increased esophageal exposure to weakly acidic reflux 5 years after laparoscopic Roux-en-Y gastric bypass. Ann Surg.

[12] Dindo D, Demartines N, Clavien PA (2004). Classification of surgical complications: a new proposal with evaluation in a cohort of 6336 patients and results of a survey. Ann Surg.

[13] Campbell J, McGarry LA, Shikora SA, Hale BC, Lee JT, Weinstein MC (2010). Cost-effectiveness of laparoscopic gastric banding and bypass for morbid obesity. Am J Manag Care.

[14] McCabe C, Claxton K, Culyer AJ (2008). The NICE cost-effectiveness threshold: what it is and what that means. Pharmacoeconomics.

[15] Kamiński B, Jakubczyk M, Szufel P (2018). A framework for sensitivity analysis of decision trees. Cent Eur J Oper Res..

[16] Husereau D, Drummond M, Petrou S, Carswell C, Moher D, Greenberg D, Augustovski F, Briggs AH, Mauskopf J, Loder E, CHEERS Task Force (2013). Consolidated health economic evaluation reporting standards (CHEERS) statement. Value Health.

[17] Li K, Zou J, Tang J, Di J, Han X, Zhang P (2016). Robotic versus laparoscopic bariatric surgery: a systematic review and meta-analysis. Obes Surg.

[18] Economopoulos KP, Theocharidis V, McKenzie TJ, Sergentanis TN, Psaltopoulou T (2015). Robotic vs. laparoscopic Roux-En-Y gastric bypass: a systematic review and meta-analysis. Obes Surg.

[19] Markar SR, Karthikesalingam AP, Venkat-Ramen V, Kinross J, Ziprin P (2011). Robotic vs. laparoscopic Roux-en-Y gastric bypass in morbidly obese patients: systematic review and pooled analysis. Int J Med Robot.

[20] Wang L, Yao L, Yan P, Xie D, Han C, Liu R, Yang K, Guo T, Tian L (2018). Robotic versus laparoscopic Roux-en-Y gastric bypass for morbid obesity: a systematic review and meta-analysis. Obes Surg.

[21] Bailey JG, Hayden JA, Davis PJ, Liu RY, Haardt D, Ellsmere J (2014). Robotic versus laparoscopic Roux-en-Y gastric bypass (RYGB) in obese adults ages 18 to 65 years: a systematic review and economic analysis. Surg Endosc.

[22] Brethauer SA, Kothari S, Sudan R, Williams B, English WJ, Brengman M, Kurian M, Hutter M, Stegemann L, Kallies K, Nguyen NT, Ponce J, Morton JM (2014). Systematic review on reoperative bariatric surgery: American society for metabolic and bariatric surgery revision task force. Surg Obes Relat Dis.

[23] Bertoni MV, Marengo M, Garofalo F, Volontè F, La Regina D, Gass M, Mongelli F (2021). Robotic-assisted versus laparoscopic revisional bariatric surgery: a systematic review and meta-analysis on perioperative outcomes. Obes Surg.

[24] Nasser H, Munie S, Kindel TL, Gould JC, Higgins RM (2020). Comparative analysis of robotic versus laparoscopic revisional bariatric surgery: perioperative outcomes from the MBSAQIP database. Surg Obes Relat Dis.

